# Penile Rehabilitation after Pelvic Cancer Surgery

**DOI:** 10.1155/2015/876046

**Published:** 2015-02-15

**Authors:** Fouad Aoun, Alexandre Peltier, Roland van Velthoven

**Affiliations:** ^1^Department of Urology, Jules Bordet Institute, 1 Héger-Bordet Street, 1000 Brussels, Belgium; ^2^Université Libre de Bruxelles, 50 Franklin Roosevelt Avenue, 1050 Brussels, Belgium

## Abstract

Erectile dysfunction is the most common complication after pelvic radical surgery. Rehabilitation programs are increasingly being used in clinical practice but there is no high level of evidence supporting its efficacy. The principle of early penile rehabilitation stems from animal studies showing early histological and molecular changes associated with penile corporal hypoxia after cavernous nerve injury. The concept of early penile rehabilitation was developed in late nineties with a subsequent number of clinical studies supporting early pharmacologic penile rehabilitation. These studies included all available phosphodiesterase type 5 inhibitors, intracavernosal injection and intraurethral use of prostaglandin E1 and to lesser extent vacuum erectile devices. However, these studies are of small number, difficult to interpret, and often with no control group. Furthermore, no studies have proven an in vivo derangement of endothelial or smooth muscle cell metabolism secondary to a prolonged flaccid state. The purpose of the present report is a synthetic overview of the literature in order to analyze the concept and the rationale of rehabilitation program of erectile dysfunction following radical pelvic surgery and the evidence of such programs in clinical practice. Emphasis will be placed on penile rehabilitation programs after radical cystoprostatectomy, radical prostatectomy, and rectal cancer treatment. Future perspectives are also analyzed.

## 1. Introduction

Cancer remains one of the leading causes of morbidity and mortality worldwide. It is predicted that, by 2020, the number of new cases of cancer in the world will increase to more than 15 million [1]. Improvements in cancer diagnosis and treatment have led to an increased life expectancy; but cancer diagnosis and treatment carry serious physical and psychological consequences that can dramatically decrease quality of life [2].

However, after the World Health Organization definition of health, attention of the scientific community shifted from the mere focus on the body and its organic affection processes to the patients and to the repercussions in patients whole being [3]. Sexual dysfunction represents a prevalent long-term complication among cancer survivors with a wide spectrum of manifestations and a huge impact on quality of life [4].

Owing to the anatomical location of the sexual organs and their innervations, radical surgery for pelvic cancers has understandably been associated with sexual dysfunction. Among men, these include erectile dysfunction (ED), penile shortenings, penile curvature, dysorgasmia, and ejaculatory disorders including retrograde ejaculation, loss of or alterations in ejaculation, and urine leakage at the time of orgasm (climacturia) [5]. In addition, relatively nonspecific problems such as changes in level of sexual activity, a lack of sexual enjoyment, and alterations in body image have been also identified in men following diagnosis or treatment of pelvic cancer [6, 7]. Moreover, sexual function remains important to men, who often continue to be interested in sex even in their final decades of life [8, 9], and ED represents the most frequent and documented sexual dysfunction after radical pelvic surgery [7]. In fact, it is estimated that 10% of all men with ED have the cause as a result of pelvic radical surgery [10]. ED was defined by The National Institutes of Health (NIH) as the “inability to attain and/or maintain a penile erection sufficient for satisfactory sexual performance” [11]. The World Health Organization and the International Consultation on Urologic Disease had also endorsed this definition [12]. In recent years, numbers of investigators have increasingly focused on ED after radical pelvic surgery. They directed their efforts toward searching for interventions that might improve sexual function. Various coping strategies and rehabilitation programs have been suggested and applied with different success rates. The rehabilitation program to increase the success rate and to shorten the interval to regain spontaneous erection is the most studied and documented program in the contemporary literature. Penile corporal hypoxia due to the loss of daily and nocturnal erections during rapid eye movement sleep leads to penile atrophy, smooth muscle apoptosis, venoocclusive dysfunction, and penile scarring and fibrosis that limit further oxygenation [13]. To break this vicious cycle, the concept of early intervention to oxygenate the penile corporal, termed penile rehabilitation, was first suggested in a paper written in 1997 by Montorsi et al. [14]. This novel idea had gained interest in many cancer centers worldwide while others remain reluctant which further points out the necessity of working out an optimal treatment strategy in order to rapidly evolve beyond the proof of concept.

The present report is a synthetic overview of the literature in order to analyze the concept and the rationale of rehabilitation program of ED following radical pelvic surgery and the evidence of such programs in clinical practice. Emphasis will be placed on penile rehabilitation programs after radical cystoprostatectomy (RC), radical prostatectomy (RP), and rectal cancer treatment.

## 2. Pathophysiology Based Concept of Penile Rehabilitation: What Is the Rationale?

The mechanisms underlying ED after radical pelvic treatment are partially elucidated and thought to be multifactorial in aetiology. In the early 1980s, Walsh et al. described a modified technique for RP in order to avoid injury of the branches of the pelvic plexus that innervate the corpora cavernosa. They evaluated retrospectively in a small case series of 12 patients who underwent a nerve sparing RP the postoperative sexual function. All of them have experienced erections and six have achieved successful vaginal penetration and orgasm. Of the six patients with sexual partners who have been followed up for 6 months or longer, five were fully potent. They demonstrated that ED was of neurogenic etiology due to cavernous nerve transaction [15]. However, regardless of the surgical technique, spontaneous erectile function was frequently impaired in the early postoperative period, probably because of a reversible injury of the cavernous nerves due to traction, exposure, or dissection, a process known as neurapraxia or transient cavernosal nerve dysfunction. This temporary deficit can abolish any form of erection and can last up to two years [16]. More recently, a vascular etiology of postsurgical ED has generated increasing interest, suggesting that loss of daily and nocturnal erections might lead to irreversible damage to the cavernous tissue. Several animal and clinical studies corroborated these findings.

In early studies, smooth muscle cell apoptosis was demonstrated as early as the first postoperative day after bilateral and unilateral cavernous neurectomy in a rat model compared to a more delayed smooth muscle cell apoptosis after cavernous nerve crush injury [17]. Smooth muscle apoptosis appeared to be clustered in the subtunical area and contributed to venous leak, after RP, when smooth muscle content in the penis drops below 40% [18]. Mulhall et al. demonstrated, by using preoperative and postoperative hemodynamic assessment, that more than half of the patients treated by RP had venous leak after surgery [19]. The incidence of venous leak ranged from 14% at 4 months to more than 50% at 12 months after nerve sparing RP according to their studies. Another consequence of neurapraxia is alteration in the smooth muscle-collagen ratio with increased levels of collagen types I and III as well as elevated levels of transforming growth factor b1 [20, 21]. These changes have previously been associated with prolonged tissue hypoxia, leading many investigators to propose a causal relationship between hypoxia and the cavernosal changes seen in men with a prolonged flaccid state after NSRP [22]. However, the role of persistent failure of cavernous oxygenation in penile fibrosis after NSRP remains a topic of debate. Critics of the hypoxia theory point out that the effects of physiologic penile hypoxia associated with a flaccid penis on cavernosal tissue remain theoretical [23]. To date, no studies have proven an in vivo derangement of endothelial or smooth muscle cell metabolism secondary to a prolonged flaccid state. Patients with known injury to the neurovascular bundles likely proceed through a continued cycle of smooth muscle cell death, leading to irreversible venoocclusive disease. Similarly, patients with preserved neurovascular bundles might demonstrate progressive fibrosis of the cavernosal tissue during the period of neurapraxia, leading to the same endpoint of venous leak. It is this latter group that is targeted with penile rehabilitation to decrease the fibrotic changes associated with the temporary period of nerve dysfunction. It had been demonstrated that prostaglandin E1 (PGE1) and phosphodiesterase type 5 inhibitors (PDE5Is) may promote the recovery of erectile function by providing cavernosal oxygenation [24, 25]. It had been also hypothesized that these medications even in the absence of an erection can induce cavernosal oxygenation and thus could be used to preserve smooth muscle after cavernous nerve injury [26]. By performing percutaneous penile biopsies at the time of RP and 6 months later, Schwartz et al. were the first to validate the early use of PDE5Is for penile rehabilitation therapy. They were able to demonstrate that early use of sildenafil after surgery may preserve intracorporeal smooth muscle content [27]. Furthermore, it had been shown that early use of PDE5Is decreased the numbers of apoptotic cells and prevented apoptotic cell death in the penis following denervation [28, 29]. Several studies have provided important animal model documentation of the benefit of PDE5 therapy for prevention of histological changes in the penis after nerve injury [30]. Chronic therapy with both long- and short-acting PDE5I can prevent corporo-veno-occlusive dysfunction and underlying histological changes induced by neurapraxia [31, 32]. It is noteworthy to mention that these medications and devices used in rehabilitation program are safe and well tolerated by patients without significant side effect profile which is demonstrated by the high rate of compliance with these rehabilitation strategies [33].

## 3. Tailoring Penile Rehabilitation according to Preoperative and Postoperative Risk Factors

Distinguishing subgroups of patients who are in need for rehabilitation from those who are not good candidates for rehabilitation is an emerging concept in penile rehabilitation following pelvic cancer surgery. It is evident that patients with preoperative erectile dysfunction not responding to pharmacological therapy should not receive penile rehabilitation following radical pelvic surgery [34, 35]. It was also demonstrated that patients who undergo a non-nerve sparing radical prostatectomy did not benefit from pharmacological rehabilitation program [36]. It was also suggested that men <55 years of age with a good preoperative erectile function do not benefit from rehabilitation program following BNSRP [37]. The same authors suggested that penile rehabilitation may be more beneficial in older patients and patients with a diminished preoperative erectile function. Briganti et al. defined three risk categories for postoperative erectile dysfunction based on preoperative characteristics [38]. The recovery of erectile function was improved with PDE5I overall in their study. The outcome of rehabilitation was similar to that of on-demand and daily PDE5I treatment in both patients at high risk (age ≥70 years or IIEF-EF score ≤10 or a Charlson comorbidity index (CCI) score ≥2) and low risk (age ≤65 years, IIEF-EF score ≥26, and CCI score ≤1). However, the daily treatment showed significantly better effect in intermediate risk patients (age 66–69 years or IIEF-EF score 11–25 or CCI score ≤1). Subsequent studies have supported the potential role of psychosocial interventions [39]. However, long-term improvements except regarding compliance with the pharmacological program had not been demonstrated [40]. This could be due to the standard method used independently from the patient personality. Other studies have attempted to assess the role of the partner in post-RP sexual dysfunction. It has been shown that a sexually functional partner is associated with better sexual outcomes after RP and that there is a strong correlation between male and female sexual dysfunction in couples where the man has undergone RP [41, 42]. Recently, Müller et al. conducted a retrospective study on 92 patients following RP to identify the predictors of successful outcome with pharmacological penile rehabilitation following RP [43]. Positive predictor factors of a successful rehabilitation program included bilateral nerve sparing radical prostatectomy, young age (<60 years), absence of vascular comorbidities, and the instauration of a rehabilitation program <6 months after RP. Another important point is the use of adjuvant therapy. The impact of adjuvant radiation therapy on the rehabilitation program is not clear and data are lacking in the literature [44]. This uncertainty stems from the fact that few studies have addressed the impact of radiation therapy on EF in post-RP patients and also from the fact that most men after RP do not have intact EF, making it difficult to determine whether adjuvant radiation therapy results in further loss of function. However, the use of androgen deprivation therapy would have debilitation sexual side effects and renders the rehabilitation program more difficult [45]. The impact of nerve sparing radical prostatectomy on incontinence is contradictory in the contemporary literature. The presence of incontinence in patients with erectile dysfunction alters significantly their quality of life but its interference with penile rehabilitation program is not clear [46, 47].

## 4. Penile Rehabilitation after Radical Prostatectomy: What Is the Evidence?

Historically, patients suffering ED after RP were observed and encouraged during the postoperative period to wait for the return of erectile function without the need for active intervention. The results of such an approach were unsatisfactory both for the patient and for the physician.

Gallina et al. showed that, after a mean follow-up of >2 years, only 35.8% of patients left untreated after open bilateral nerve sparing radical prostatectomy (NSRP) recovered from erectile function after surgery [37]. Montorsi et al. were the first to demonstrate in a prospective study the benefits of a rehabilitation program with PGE1 in increasing the recovery rate of spontaneous erections after NSRP [14]. However, preoperative erectile function had not been assessed in their studies and no validated questionnaire had been used. Furthermore, the long-term benefit was not evaluated due to short follow-up [14]. Mulhall et al. demonstrated in a nonrandomized study the benefit of a rehabilitation program with PDE5I or ICI for nonresponders to PDE5I for patients with functional preoperative erections undergoing RP [48]. Bannowsky et al. specified that sildenafil was significantly active in cases of early postoperative nocturnal erections [49]. The findings obtained with the small patient sample of Padma-Nathan et al. showed that nightly sildenafil administration for 9 consecutive months, beginning 1 month postoperatively, resulted in a greater return to baseline erectile function [50]. These findings had also been confirmed, in clinical studies, for the other PDE5Is [51–53]. In a large contemporary series of patients treated by high-volume surgeons, the 3-year EF recovery rates were shown to be significantly higher in patients who did use postoperative PDE5Is compared with patients who did not use any postoperative PDE5Is (73% and 37%; *P* < 0.001) [54]. Intraurethral alprostadil had also been used in rehabilitation programs.

Raina et al. have treated 56 men with intraurethral alprostadil (MUSE, Vivus Inc., Mountain View, California, USA) at doses of 125 and 250 mg three times weekly for 9 months. Although MUSE therapy avoids the needles associated with ICI, it is notable that almost one-third of men did not complete the study. Treatment was initiated 3 weeks postoperatively, and 40% of patients using MUSE reported having natural erections sufficient for vaginal intercourse [55]. This noncompliance rate indicates beside side effect disorders that men need encouragement to continue with therapies that may not have immediate results. The same authors reported on the use of vacuum erection device (VED) as a rehabilitation therapy [56]. However, the results were inconclusive. Köhler et al. reported higher rates in a group of patients treated as early as 1 month with VED compared to a control group treated 6 months later with VED [57]. Further studies for VED as a rehabilitation therapy are needed especially because the mechanism of improving erectile function is unknown. The timing of rehabilitation is controversy in the literature. However, a general agreement is based on experimental studies stresses that any form of rehabilitation should begin as close to the surgery as possible. Moskovic et al. described a “massive attack” rehabilitation program where all the mentioned modalities were used even beginning one week prior to surgery [58]. In their studies, preoperative female partner sexual function correlated with greater patient compliance with the localized component of the ED rehabilitation program [58]. Despite the increased demand of rehabilitation program, a national survey in France found that only 38% of French urologists who responded to the survey prescribed systematically postoperative penile rehabilitation [59]. Another survey among the members of the International Society for Sexual Medicine showed that some form of postoperative penile rehabilitation was performed in clinical practice: 95% used PDE5I, 75% ICI, 30% VED, and 9.9% intraurethral prostaglandin [60]. Although cost represented the most common reason for rehabilitation neglect, 25% were reluctant because they were not familiar with the concept and another 25% because of lack of evidence supporting penile rehabilitation [60]. Some physicians used rehabilitation program with on-demand intake of PDE5I in order to reduce the cost. These physicians based their intervention on a randomized, double-blind, multicenter, parallel group study comparing 9 months nightly dosing of vardenafil and flexible-dose on-demand vardenafil in patients who had a bilateral NSRP [61]. Nightly dosing with vardenafil did not have any effect beyond that of on-demand use. Even more clinically relevant is the fact that this study confirmed that vardenafil taken when needed during the double-blind treatment period was associated with significantly better results compared with placebo. Finally, in a prospective, randomized, open-label, multicenter study there were no statistically significant differences between nightly intraurethral alprostadil and oral sildenafil started <1 month and continued for 9 consecutive months in potent men who underwent bilateral NSRP [62].  Table 1 summarizes the different studies on penile rehabilitation following RP.

## 5. Penile Rehabilitation after Radical Cystoprostatectomy: What Is the Evidence?

ED is a common problem reported in up to 94% of patients after RC [63]. Erectile function recovery after RC ranged between 14% and 80% [64, 65]. Although tailoring the surgical approach, such as nerve sparing RC, might improve outcomes according to recent studies, rehabilitation programs were found to be necessary to optimize recovery from erectile function [66]. The major problem stems from patients not complaining about sexual activity after this morbidly body image modifying operation. Studies have shown that the psychological and stress impact of such an operation is substantial and patients begin to complain of ED in a median of 12 to 18 months after the operation [67]. Rehabilitation begins by informing sexually active patients before the operation of the possibility of regaining sexual activity after the operation. Psychological support aims to help the couple's relationship and reassure the patient's corporeal image, while taking into consideration the impact of the surgical procedure and the diagnosis of cancer because physical and emotional disturbances are common in these settings. Attempts to preserve the neurovascular bundles without compromising cancer control are encouraged in such patients. Schoenberg et al. demonstrated that, following nerve sparing RC for organ-confined cancer, the disease specific 10-year survival rate for all stages treated was 69% and the 10-year survival rate and freedom from local recurrence were 94% [68]. Sexuality preserving cystectomy was another concept developed by Horenblas et al. The operation consists of pelvic lymph node dissection followed by cystectomy and neobladder alone, with preservation of the vas, prostate, and seminal vesicles. The authors reported that 7/10 men who underwent surgery with this modification had return of normal erections on Doppler ultrasound measurement [69]. In a later update, this group reported that 20/24 males were sexually active with or without erect aids and concluded that preservation of the prostate and neurovascular pedicle led to improved sexual function [70]. However, there are urodynamic concerns leaving the prostate since the obstruction may occur secondary to weak propulsive pressure of the neobladder. In 2002, Vallancien et al. proposed an alternative nerve sparing cystectomy approach, which includes a TURP before cystectomy [71]. The principles include a TURP leaving the prostatic capsule with nerve sparing cystectomy, with preservation of seminal vesicles and vas deferens. In 61 sexually active preoperative patients, they reported that 50 (82%) had maintained their potency after a mean follow-up of 3.8 years. The oncologic concern with these procedures is that prostatic urethra and seminal vesicles can be a potential site for recurrence. For these reasons, sexuality preserving cystectomy is not recommended by urologic societies. To date, there are few studies addressing penile rehabilitation after RC. Hautmann et al. reported on 9 patients undergoing nerve sparing RC [72]. Four out of nine patients maintained spontaneous complete tumescence, and five patients had partial tumescence using sildenafil as a successful rehabilitation strategy. Zippe et al. evaluated the effect of sildenafil citrate intake on erectile function after RC; 42 out of 49 patients did not have erections sufficient for vaginal penetration and 22 out of these 42 patients were given sildenafil citrate but only 2 patients (9%) responded positively [63]. However, El-Bahnasawy et al. reported higher rates of response with the use of full dose sildenafil. Vaginal penetration was possible in 33% and 54.2% of patients taking 50 and 100 mg of sildenafil, respectively. The ability to maintain the erections after penetration was reported in 29% and 53.1% of cases with 50 and 100 mg of sildenafil, respectively [73]. In RC series, the efficacy of sildenafil increased significantly on increasing the dose to 100 mg [73]. Furthermore, these studies show a trend toward a better response to the rehabilitation program for continent patients and patients with orthotopic bladder reconstruction compared to incontinent patients and patients with other forms of urinary diversion [73]. This finding can be explained by the fact that orthotopic diversion is usually offered to generally healthy patients with confined tumors located away from the bladder neck. Moreover, during RC, particular attention is directed to preparing a good urethral stump and maintaining good sphincter action, and this usually needs meticulous dissection and preservation of the neurovascular bundles.

## 6. Penile Rehabilitation after Rectal Surgery: What Is the Evidence?

ED is a prevalent problem reported in 10 to 60% of patients undergoing rectal cancer surgery [74]. In a historic prospective case series study, ED was found in 48% of patients after abdominoperineal resection (APR) [75]. APRs carry a higher risk of postoperative ED than low anterior resection procedure with reported rates varying from 15 to 92% but ED rates as high as 73% were reported after low anterior resection [76, 77]. The permanent colostomy made after APR has also been shown to alter the body image and increases the rate of postoperative sexual dysfunction but there is also controversy in small series showing no difference between patients with or without a colostomy [78]. Patients younger than 50 years had a minimal risk of ED [79]. Surgical expertise is another factor potentially affecting ED rates with case series from high surgeon volume and high cancer center volume, reporting lower rates of ED [80]. Rehabilitation programs for these patients are complex and often require a pluridisciplinary approach. Specifically trained health care personnel are always necessary. Psychological evaluation and support of the patient and his/her partner are mandatory [81]. Moreover, they can enhance the response to pharmacologic therapy [81]. Among the medications available, the efficacy of sildenafil was demonstrated in a study where 32 patients who had undergone rectal resection were randomized to medical treatment or placebo [82]. Erectile function improved in 80% of patients treated with sildenafil compared to 17% of patients treated with placebo [82]. Nowadays, most experienced surgeons are currently performing total mesorectal excision (TME) with preservation of the neurovascular bundles with improved reported rates of ED [83–85]. However, only one prospective study conducted in Japan examined the outcome of postoperative treatment with sildenafil for sexually active patients treated by total mesorectal excision (TME) for low rectal cancers [78]. 40 out of 49 sexually active patients preoperatively presented ED at 3 months postoperatively, and only 4 patients regained their erection at 12 months. Sildenafil was administered to 16 patients who requested the drug during follow-up, and sexual dysfunction was improved in 11 of these patients. Sildenafil has also been reported to improve anal function [86, 87] but further experimental researches are needed to understand the mechanism of action and its impact on postoperative anal function.

## 7. Future Perspectives: The Next Decades of Treatments

The understanding of nerve injury and nerve regeneration and its treatments will be an exciting research area in the next decade. Accumulating evidence suggests that a return of potency following radical pelvic surgery is partially dependent on regeneration of the cavernous nerves. In reconstructive surgery, it had been demonstrated that nerve regeneration occurs at a rate of 1–3 mm/day depending on the age of the patient as well as other regional factors [88]. Recent interest has focused on neuromodulation strategies that can minimize nerve injury, promote nerve regeneration, and/or protect endothelium and cavernosal smooth muscle. Several treatment strategies directed against inflammation, immunologic reactions, ischemic changes, free radical production, lipid peroxidation, and apoptosis have been under investigation in animal models with preliminary promising results for some agents. In a rat model, Lagoda et al. showed that concomitant administration of immunophilin ligands with PDE5I improved the recovery of erectile function following cavernous nerve injury [89]. The only clinical study in humans was conducted by Burnett and colleagues. This multi-institutional, randomized, placebo controlled study failed to detect neuroprotective benefit from the immunophilin ligand [90]. In contrast, erythropoietin injection improved postoperative erectile function in a clinical retrospective study published by the same authors [91]. Recently, the role of vascular growth factors in promoting the regeneration of damaged cavernous nerves and return of erectile function has been investigated in animal models. Intracavernous administration of vascular endothelial growth factors (VEGF) facilitates the recovery of nitric oxide synthase genes, which may promote the earlier recovery of sexual function [92]. Lee et al. from the same group later reported that intracavernous injection of VEGF increases the recovery of erectile function in the rat models [93]. Cavernosal nerve reconstruction using genitofemoral or sural nerve has generated a considerable interest in an attempt to preserve the erectile function but its current use is limited to young patients with extensive radical surgery in high-volume centers [94, 95]. Nerve growth factor and fibroblast growth factor with combination of nerve grafts are also used with encouraging results in animal models [96]. The use of intracorporeal vectors that can replace damaged endothelial cells and promote the nitric oxide synthesis represents an exciting model for the future research [97]. Recently, the area of interest is shifted to nitric oxide donors, drugs that increase the nitric oxide synthesis in the cavernosal bodies. To date, two agents (NCX 4050, NCX 911) have demonstrated in vitro and in vivo increase in smooth muscle relaxation [98, 99]. Lastly, injection of mesenchymal stem cells into the corpus cavernosum has caused enormous excitement but much more scientific investigation is needed before implementing this therapy in daily rehabilitation practice.

## 8. Conclusion

Erectile dysfunction is the most frequent complication after pelvic radical surgery. Its negative impact on the quality of life among cancer survivors is well documented in the literature. However, technical advances and molecular progress have modified the natural history of postoperative erectile dysfunction. Rehabilitation programs for patients undergoing pelvic radical surgery are complex and often require a pluridisciplinary and multistep approach. Evaluating erectile function and sexual activity of the patient and his/her partner before the operation is an integral part of the rehabilitation strategies. Patients are informed about the potential risk of ED and about the potential benefits of an early penile rehabilitation. Tailoring the surgical procedure in accordance with patient characteristics and tumor spread is the most important step in preventing unnecessary neurologic damage. Specifically trained health care personnel are necessary. Psychological evaluation and support of the patient and his/her partner in a family based intervention approach can enhance the response to early pharmacologic therapy. Oral PDE5Is are used on daily or on-demand schedule with timing as close to the operation as possible. In case of nonresponse, intracavernosal injection or intraurethral use of PGE1 is tried, alone or in combination with PDE5I, two to three times per week. Close and long-term follow-up is needed to encourage and observe these patients. Recent neuromodulation strategies, nerve reconstruction, and stem cell use with the potential to minimize nerve injury, promote nerve regeneration, and/or protect endothelium and cavernosal smooth muscle are, nowadays, limited to high-volume research center but could be integrated in future rehabilitation programs.

## Figures and Tables

**Table 1:** Summary table of penile rehabilitation trials in patients undergoing pelvic cancer surgery.

Author (year of publication)	Study design	Type of pelvic cancer surgery	Number of patients	Penile rehabilitation modality	Active drug (dosage)	Regimen	Duration	Outcomes
Montorsi et al. (1997) [14]	PRCT	NSRP	30	ICI	Alprostadil (2.5–14 *µ*g)	3 times weekly starting 1 month after surgery	12 weeks	Recovery of spontaneous EF
Mulhall et al. (2005) [48]	Prospective controlled nonrandomized trial	RP	132	PDE5I or ICI for nonresponders to PDE5I	Sildenafil (50–100 mg) and alprostadil	Daily oral PDE5I or 3 times weekly ICI for nonresponders	18 months	2.7 times higher spontaneous EF and statistically higher IIEF scores
Bannowsky et al. (2008) [49]	PRCT	NSRP	41	PDE5I	Sildenafil (25 mg)	Nightly low dose starting the day of catheter removal	52 weeks	Higher spontaneous EF and statistically higher IIEF scores
Padma-Nathan et al. (2008) [50]	PRCT	BNSRP	76	PDE5I	Sildenafil (50–100 mg)	Nightly starting 4 weeks after surgery	36 weeks	Improvement in spontaneous EF and satisfaction
Montorsi et al. (2004) [34, 51]	PRCT	BNSRP	303	PDE5I	Tadalafil (20 mg)	On demand 12 to 48 months after surgery	12 weeks	Statistically higher IIEF scores and higher satisfaction
Brock et al. (2003) [52]	PRCT	NSRP	440	PDE5I	Vardenafil (10–20 mg)	On demand	12 weeks	Statistically higher IIEF scores
Raina et al. (2006) [100]	PRCT	NSRP	109	VED	VED	Daily starting two weeks after surgery	9 months	Improvement in spontaneous EF, IIEF scores, and satisfaction
Raina et al. (2007) [55]	Prospective controlled nonrandomized trial	NSRP	91	Transurethral	MUSE (125 or 250 *µ*g)	3 times weekly starting 3 weeks after the surgery	9 months	Recovery of spontaneous EF
Köhler et al. (2007) [57]	PRCT	NSRP	28	VED	VED	Daily (10 mins) (immediate (1 month) versus delayed (6 months))	5 months	Improvement of EF and preservation of penile length
Montorsi et al. (2008) [61]	PRCT	BNSRP	628	PDE5I	Vardenafil (10 mg nightly versus 5/20 mg on demand)	10 mg nightly versus 5/20 mg on demand	9 months	No difference in IIEF-EF between nightly dosing and on-demand dosing
McCullough (2008) [30]	PRCT	NSRP	54	Transurethral versus PDE5I	MUSE (125 *µ*g) versus sildenafil (50 mg)	Nightly starting 1 month after the surgery	9 months	No differences in recovery
Schwartz et al. (2004) [27]	Prospective controlled nonrandomized trial	NSRP	21	PDE5I	Sildenafil (50 mg versus 100 mg)	Every other night beginning the day of catheter removal	6 months	No loss of smooth muscle in 50 mg and gain of smooth muscle in 100 mg
Nandipati et al. (2006) [101]	Prospective controlled nonrandomized trial	NSRP	22	PDE5I and ICI	Sildenafil (50 mg) and alprostadil (1–4 *µ*g) or trimix (20 U)	Sildenafil daily and ICI 2-3 times weekly at hospital discharge	6 months	Assisted early sexual activity and satisfaction; addition of PDE5I allows lower dose of ICI
Zippe et al. (2004) [63]	Retrospective	RC	49	PDE5I	Sildenafil (?)	Not specified	Not specified	Successful vaginal penetration in 9% of patients
Hautmann et al. (2010) [72]	Retrospective	RC	9	PDE5I	Sildenafil (?)	Not specified	Not specified	Partial tumescence in 5/9 patients
El-Bahnasawy et al. (2008) [73]	Prospective nonrandomized trial	RC	100	PDE5I	Sildenafil (50–100 mg)	Daily	4 weeks with 50 mg and then 4 weeks with 100 mg	Dose related effect
Nishizawa et al. (2011) [78]	Prospective nonrandomized trial	Rectal cancer surgery	49	PDE5I	Sildenafil (25 mg) and vardenafil (5 mg) or sildenafil (50 mg) and vardenafil (10 mg)	On demand	Not specified	Improvement in EF in 69% of patients
Lindsey et al. (2002) [82]	PRCT	Rectal cancer and inflammatory bowel disease surgery	32	PDE5I	Sildenafil (25–50–100 mg)	Dose escalation	4 weeks	79% responded to sildenafil, on global efficacy assessment, compared with 17% taking placebo (*P* = 0.0009)

PRCT: prospective randomized controlled trial; PDE5I: phosphodiesterase type 5 inhibitor; ICI: intracavernous injection; MUSE: medicated urethral system erection; RP: radical prostatectomy; RC: radical cystectomy; NSRP: nerve sparing radical prostatectomy; BNSRP: bilateral nerve sparing radical prostatectomy; EF: erectile function; IIEF: international index of erectile function; IIEF-EF: erectile function domain of the international index of erectile function.
